# Assessment of sugar-sweetened beverage consumption and weight change: a prospective cohort study

**DOI:** 10.1186/s40795-017-0182-y

**Published:** 2017-08-18

**Authors:** Patrick Mullie, Philippe Autier, Mathieu Boniol, Peter Boyle, Benedicte Deforche, Evelien Mertens, Ruben Charlier, Sara Knaeps, Johan Lefevre, Peter Clarys

**Affiliations:** 1 International Prevention Research Institute, 95 Cours Lafayette, 69006 Lyon, France; 2 Department of Human Biometrics and Biomechanics, Faculty of Physical Education and Physiotherapy, Vrije Universiteit Brussel, Pleinlaan 2, 1050 Brussels, Belgium; 3 Erasmus University College, Laarbeeklaan 121, 1120 Brussels, Belgium; 4 International Prevention Research Institute, University of Strathclyde Institute of Global Public Health at iPRI, 95 Cours Lafayette, 69006 Lyon, France; 5 Department of Public Health, Faculty of Medicine and Health Sciences, Ghent University, De Pintelaan 185, 9000 Ghent, Belgium; 6 Department of Kinesiology, KU Leuven, Faculty of Kinesiology and Rehabilitation Sciences, Tervuursevest 101, 3001 Leuven, Belgium; 7 Department of Movement and Sports Sciences, Ghent University, Watersportlaan 2, 9000 Ghent, Belgium

**Keywords:** Nutritional assessment, Obesity, Sugar-sweetened beverages, Weight gain

## Abstract

**Background:**

In prospective studies, sugar-sweetened beverages (SSB) have been associated with weight increase. However, most prospective studies examine changes in body weight over time according to amounts of SSB intake at baseline, generally without considering changes over time in SSB, energy intake and physical activity. The objective of the present study was to examine how SSB intakes influence changes in weight, according to the way SSB intakes are analysed.

**Methods:**

For a prospective cohort study with two nutritional assessments in time, 46 Flemish municipalities were selected by clustered random sampling. Within these municipalities, a random sample of men and women between 18 and 75 years of age was selected and invited to participate. In total, 562 middle-aged Belgian adults were tested in 2002 and 2012 for the same anthropometric, lifestyle, nutrition and physical activity parameters. The main outcome measured were weight changes from 2002 to 2012 were analysed according to various ways to parametrise SSB intakes in 2002 and changes in SSB, energy intake, and physical activity from 2002 to 2012.

**Results:**

In a multivariable model including age, sex, the best predictors of weight changes were the weight at baseline (*P* < 0.001), then the change in energy intake (*p* = 0.068). No association was found with SSB intake at baseline (*P* = 0.267) and the change in SSB intake (*P* = 0.130).

**Conclusions:**

Results of prospective studies on SSB intake and body weight depend much on the way SSB intakes are analysed, and on the inclusion of changes in energy intake and physical activity in analyses.

## Background

The relation between added sugar, sugar-sweetened beverages (SSB) consumption and weight gain has been the subject of a number of observational and intervention studies. The most comprehensive meta-analysis published to date by Te Morenga et al. found that in prospective observational studies, subjects in the highest categories of SSB intake had a 55% greater probability (95% CI: 1.32 to 1.82) to be overweight or obese than subjects in the lowest categories of SSB consumption [1]. The meta-analysis also found that in intervention studies with ad libitum energy intake, the highest consumption category of added sugar intake, a main component of SSB, was associated with a mean weight gain of 0.75 kg (95% CI: 0.30 kg to 1.19 kg) at the end of the intervention. In contrast, intervention studies in which added sugars were exchanged against an iso-energetic amount of other food showed no weight gain associated with increasing added sugar consumption. The contrast between iso-energetic and ad libitum intervention studies indicates that the influence of added sugar in SSB on weight gain would essentially be the energy content and not some physiological effect that would be independent of thermodynamic properties [2].

This contrast also points to the limitations of observational studies on sugar intake, SSB consumption and adiposity. Meta-analyses of prospective observational studies are mostly based on a comparison of changes in body weight (or another maker of adiposity) over time between groups of subjects with the highest and the lowest categories of sugar or SSB consumption. This procedure mixes together studies whose most extreme categories of exposure refer to variable amounts of sugar consumption. Moreover, it ignores how these most extreme exposure categories were formed, i.e., for instance whether they represent the highest and lowest quantiles (e.g., a category for no intake, and then amounts consumed in quartiles) or the way authors decided about category boundaries. Most studies assessed the intake of sugar and/or SSB at baseline only, and their results were thus based on the implicit assumption that changes in the intake of sugar and/or SSB during the 2 to 6 years follow-up had no influence on body weight changes.

In this cohort study, the assessment of food and beverage intakes were done at baseline in 2002 and ten years later in 2012 using the same three-day food record. We prospectively examined how SSB intakes influence weight changes, according to the way SSB intakes are analysed, and according to adjustments for energy and physical activity. We analysed the data in several ways which mimicked analyses done in prospective and intervention studies.

## Methods

### Subjects

Data were collected by the Flemish Policy Research Centre Sport, Physical Activity and Health [3]. One aim of the Research Centre was to investigate the relationship between nutritional habits, physical health, mental health and physical fitness in an adult population. For this purpose, 46 Flemish municipalities were selected by clustered random sampling. Within these municipalities, a random sample of men and women between 18 and 75 years of age was selected and invited to participate. Detailed establishment and description of this sample have been given elsewhere [4]. Subjects were asked to visit the central test laboratory to have anthropometric measurements taken and to complete questionnaires. A three-day food record was sent about two weeks before their visit to the laboratory, and subjects were requested to bring their completed record on the day of their appointment. The first visit to the test laboratory took place in 2002, and the second visit took place in 2012. Of the original 1569 participants in 2002, 562 (36%; 367 men and 195 women) returned for participation in 2012. In general, men and women from the follow-up study had a lower adiposity than the drop-outs [5]. The study was approved by the ethical and medical committee of the Catholic University of Leuven, Belgium. All participants signed an informed consent form and received information about the tests and measurements.

### Dietary assessment

Participants noted all foods and drinks consumed during two non-consecutive weekdays and one weekend day. The participants were instructed to weigh the amount of foods and drinks consumed. If weighing was not possible, the participants were instructed to estimate the amount of the foods and drinks they consumed by using standard household measures (e.g. a spoon, glass, cup, etc.). All information about the food record was included in the three-day record booklet. Diet records were analysed using the Becel Nutrition software (Unilever Co.; Rotterdam, The Netherlands). Total energy intake (in kcal/day) and macronutrients (in gram/day) were calculated using quantities of foods and beverages consumed. We defined SSB as beverages containing added caloric sweeteners such as sucrose and/or high-fructose corn syrup.

From the nutritional data, two a-priori dietary pattern scores were derived, the Healthy Eating Index and the Mediterranean Diet Score. Both were computed as described before [6, 7]. The possible scores ranged from 0 to 100 for Healthy Eating Index and for Mediterranean Diet Score, with a high score denoting a high adherence to a diet.

### Anthropometric measurements

Anthropometric measurements were performed by trained staff using standardized techniques and equipment according to the International Society for the Advancement of Kinanthropometry [8]. Participants were measured barefoot and in minimal clothing. Body weight was recorded to the nearest 0.1 kg with a digital balance (Seca 841, Seca GmbH, Hamburg, Germany) and body height with a Holtain stadiometer (Holtain, Crymych, UK) to the nearest 0.1 cm. BMI was calculated using the following formula: BMI = body weight (kg)/(height (m))^2^.

### Physical activity level (PAL)

The PAL was estimated using the Flemish Physical Activity Questionnaire (FPACQ). The FPACQ was found to be a valid and reliable questionnaire for the different components of physical activity during a normal week in adults [9]. The PAL is an indicator of the general activity level expressed in relation to the basal metabolism. A PAL between 1.40 and 1.69 is defined as sedentary, between 1.70 and 1.99 as moderately active and from 2.00 onwards as vigorously active [9].

### Smoking behaviour

Smoking behaviour was assessed using the WHO Monica Smoking Questionnaire that enabled dichotomization in current smokers and current non-smokers [10].

### Statistical analysis

SPSS 21.0 (SPSS Inc. Chicago, IL) statistics software was used for data analysis. Chi-square tests and paired samples t-tests were used for characterization of the participants and to examine differences in BMI, weight, physical activity and nutrition between the two periods (2002 and 2012). Weight change was categorized in weight increase of one kg or more, weight decrease of one kg or more, and stable weight. Weight and BMI differences were calculated by subtracting weight and BMI values at the second visit minus the value at the first visit.

The SSB consumption had a non-normal distribution, with a high number of non-consumers. Four categories of baseline SSB consumption were created, i.e. no consumption, low daily consumption (less than one glass or 200 ml/d), moderate daily consumption (between one and three glasses or 200 and less than 700 ml/d) and high daily consumption (more than three glasses or 700 ml/d and more). Distribution of characteristics of participants between consumption categories were tested by ANOVA. A two-sided 0.05 level of significance was defined.

For examining the influence of factors on weight changes that took place from 2002 to 2012, we defined a multivariable linear regression model with weight in 2012 as the dependent variable and sex, age and weight in 2002 included in the model as a continuous independent variable. Two types of models were then further elaborated: a first type that consisted in the base model with addition of values of physical activity, sugar-sweetened beverages intake and total energy intake in 2002. The second type of model was the base model with addition of the residual values of physical activity, sugar-sweetened beverage intake and total energy intake.

Residual values were created by regression of the measures done in 2012 onto the measures done in 2002. The residual values can be interpreted as the amount of change between the first (2002) and second (2012) visit to the test laboratory. These residual values are independent of values found at first visit in 2002. Residual values are preferable to changes in exposure category over time because they are less affected by auto-correlated error and regression to the mean effects [11].

When SSB consumption was included in models as a continuous variable, a logarithmic transformation was applied in order to soften the skewness of the distribution. In all models, energy intake was expressed in units of 40 kcal, which is the energy content of 10 g of sugar.

## Results

Exposure and outcomes were assessed in 2002 and 2012 in 562 middle-aged adults (367 men and 195 women) (Table 1). Over the 10-year period, men and women gained a mean (SD) of 1.0 (5.2) and 1.8 (4.8) kg on average, respectively. However, these averages do not reflect well individual weight changes. While about half men and women experienced weight increase during the decennium, one quarter of men and one fourth of women experienced weight decrease. Physical activity remained stable in women, but decreased significantly in men. Smoking rates reduced from 13.4% to 8.7% in men and from 13.3% to 8.2% in women.

**Table 1:** Description of the participants to the test periods of 2002 and 2012

	2002				2012				*P* for men	*P* for women
	men (*n* = 367)		women (*n* = 195)		men (*n* = 367)		women (*n* = 195)			
	mean	SD	mean	SD	mean	SD	mean	SD		
Age	46.9	8.4	45.3	8.4	57.4	10.2	55.8	8.4		
BMI (kg/m^2^)	25.4	2.7	23.4	3.0	25.7	3.0	24.1	3.4	<0.001^a^	<0.001^a^
Weight (kg)	79.3	10.1	63.9	8.7	80.4	10.8	65.7	9.7	<0.001^a^	<0.001^a^
BMI difference (kg/m^2^)					0.3	1.6	0.6	1.7		
Weight difference (kg)					1.0	5.2	1.8	4.8		
Physical activity level^c^	1.8	0.2	1.7	0.2	1.7	0.3	1.7	0.2	0.003^a^	0.297^a^
	n	%	n	%	n	%	n	%		
BMI below 25.0 kg/m^2^	173	47.1	136	69.7	157	42.8	131	67.2	<0.001^b^	<0.001^b^
BMI between 25.0 and 29.9 kg/m^2^	177	48.2	50	25.6	178	48.5	53	27.2		
BMI more than 30.0 kg/m^2^	17	4.6	9	4.6	32	8.7	11	5.6		
Weight decrease					118	32.2	49	25.1		
Weight stable					73	19.9	41	21.0		
Weight increase					176	48.0	105	53.8		
Current smoking	49	13.4	26	13.3	32	8.7	16	8.2	<0.001^b^	<0.001^b^

*BMI* body mass index, *SD* standard deviation; BMI, and weight difference = measure_2012 - measure_2002; Physical activity level was estimated using the Flemish Physical Activity Questionnaire

^a^ *P*-value according to paired t-test

^b^ *P*-value according to chi-square test

^c^Physical activity level was estimated using the Flemish Physical Activity Questionnaire [9]

From 2002 to 2012, the mean energetic intake decreased from 2590 to 2421 kcal/d for men and from 2026 to 1958 kcal/d for women (Table 2). Mean total sugar intake decreased from 88 to 80 g/d in men and from 69 to 64 g/d in women. Total fat intake remained stable during this period, and alcohol intake increased in men. Between 2002 and 2012, SSB consumption decreased in men from 4.8 energy-percent to 3.2 energy-percent. This was for women 2.4 and 1.6 energy-percent. In 2002, 40% of men and 34% of women consumed SSB. Ten years later, these proportions were significantly lower at 22% and 15%, respectively.

**Table 2:** Average nutritional intake calculated from the three-day food records

	Unit	2002				2012			
		men		women		men		women	
		mean	SD	mean	SD	mean	SD	mean	SD
Total energy	kcal	2590^a^	675	2026	499	2421^a^	626	1958	542
	kJ	10,848^a^	2825	8482	2089	10,139^a^	2623	8200	2270
Proteins	g	98.2^a^	25.7	79.9	22.0	93.3^a^	25.2	77.0	18.6
	%	15.5	3.3	16.0	3.3	15.8	3.7	16.2	3.6
Carbohydrates	g	296.5^a^	89.8	228.3	64.6	272.1^a^	82.8	218.5	69.2
	%	45.9	7.5	45.2	6.7	45.0	7.8	44.7	7.4
Total sugar	g	88^a^	45	69	35	80^a^	42	64	35
	%	13.5	5.9	13.7	5.6	13.1	5.7	13.0	5.8
Total fat	g	100.0^a^	37.4	80.3	29.1	93.5^a^	35.6	78.8	30.3
	%	34.2	7.0	35.2	7.0	34.2	6.9	35.5	6.9
Saturated fat	g	39.5^a^	15.6	32.0	12.4	36.7^a^	15.0	30.4	13.3
	%	13.5	3.2	14.0	3.2	13.4	3.2	13.6	3.4
Mono-unsaturated fat	g	38.2^a^	15.4	30.0	12.1	35.6^a^	15.1	29.7	12.3
	%	13.1	3.4	13.2	3.5	13.0	3.4	13.4	3.4
Poly-unsaturated fat	g	16.9	7.6	13.2	6.2	16.1	7.1	13.7	6.5
	%	5.8	2.0	5.8	2.0	5.9	1.9	6.3	2.2
Alcohol	g	16.3	16.2	10.0	11.9	17.2	16.6	9.6	10.8
	%	4.5^a^	4.6	3.6	4.4	5.2^a^	5.1	3.5	4.0
SSB	ml en-%	129 4.8	240 8.7	51 2.4	151 6.7	83 3.2	198 7.2	31 1.6	107 6.4
Consumers of SSB	n, %	148 (40)		43 (22)		123 (34)		30 (15)	

*En-%* energy-percent, *SSB* sugar sweetened beverages

^a^ *P* < 0.01 for 2002 versus 2012; paired t-test

We classified subjects according to their consumption of SSB in 2002 (Table 3), with about two-third of subjects who did not drink SSB and 3% of subjects who were heavy SSB drinkers in 2002. Increasing SSB consumption in 2002 was significantly associated with younger age (*P* < 0.001), progressive decreases of the Healthy Eating Index (*P* < 0.001) and the Mediterranean Diet Score (*P* = 0.03). There were 12% current smokers among the low SSB categories and 18% in the moderate and high SSB categories (chi-square, *P* = 0.024). The energy intake in 2002 and 2012 steadily increased with SSB consumption and these increases also concerned caloric foods and beverages other than SSB. The mean (SD) weight increase was much more marked in subjects with high SSB intake in 2002, 6.5 (7.5) kg compared to 1.0 (5.0) kg for non-consumers. The large SDs of weight differences reflect the variability in changes over time, but most (15 of 18) subjects in the high SSB category gained weight.

**Table 3:** Nutritional and lifestyle characteristics according to SSB consumption

	Units	Categories of SSB consumption at baseline				*P* value^a^
		Never SSB	Low SSB	Moderate SSB	High SSB	
Participants	n (%)	371 (66)	82 (15)	91 (16)	18 (3)	
SSB consumption 2002	mean (SD) ml/d	0 (0)	103 (42)	336 (122)	1008 (259)	<0.001
Classified in same SSB category 2002 and 2012	%	82	20	30	33	
Move to category of greater SSB consumption in 2012	%	18	21	0	0	
Move to category of lower SSB consumption in 2012	%	0	60	70	67	
Men	%	59	70	84	83	<0.001^c^
Age 2002	mean (SD) years	47.7 (9.3)	45.7 (8.9)	43.3 (10.1)	36.9 (9.3)	<0.001
Smokers 2002	%	11	16	21	17	0.66^c^
Healthy Eating Index 2002	mean (SD)	49 (10)	45 (9)	40 (9)	33 (5)	<0.001
Mediterranean Diet Score 2002	mean (SD)	3.9 (1.5)	3.9 (1.5)	3.6 (1.5)	2.9 (1.2)	0.03
Physical activity level 2002^b^	mean (SD)	1.7 (0.2)	1.8 (0.2)	1.8 (0.3)	1.8 (0.3)	0.004
Physical activity level 2012^b^	mean (SD)	1.7 (0.2)	1.7 (0.2)	1.8 (0.3)	1.9 (0.3)	<0.001
Physical activity difference 2012 minus 2002^b^	mean (SD)	−0.03 (0.20)	−0.04 (0.21)	−0.04 (0.27)	0.09 (0.27)	0.03
Energy consumption 2002	mean (SD) kcal/d	2308 (653)	2429 (635)	2552 (646)	3215 (784)	<0.001
Energy consumption 2002 without SSB	mean (SD) kcal/d	2308 (653)	2384 (635)	2404 (640)	2772 (821)	0.02
Energy consumption 2012	mean (SD) kcal/d	2186 (640)	2328 (666)	2399 (532)	2781 (592)	<0.001
Energy consumption 2012 without SSB	mean (SD) kcal/d	2186 (640)	2292 (674)	2356 (519)	2605 (599)	0.003
Energy difference 2012 minus 2002	mean kcal/d	−122	−101	−153	−434	0.257
Energy difference 2012 minus 2002 without SSB	mean kcal/d	−122	−91	−48	−166	0.688
Weight 2002	mean (SD) kg	72.9 (11.7)	75.0 (11.3)	77.2 (13.4)	74.9 (13.1)	0.015
Weight 2012	mean (SD) kg	73.9 (12.2)	76.3 (11.7)	78.4 (13.8)	81.4 (13.4)	0.008
Weight difference 2012 minus 2002	mean (SD) kg	1.0 (5.0)	1.3 (4.5)	1.2 (4.8)	6.5 (7.5)	<0.001
Subjects who gained weight	n (%)	176 (47)	43 (52)	47 (52)	15 (83)	
Subjects with stable weight	n (%)	80 (22)	16 (20)	16 (18)	2 (11)	
Subjects who lost weight	n (%)	115 (31)	23 (28)	28 (31)	1 (6)	

*SSB* sugar sweetened beverages

Categorization for SSB consumption in 2002: never SSB; low SSB consumption (less than 200 ml/d); moderate SSB consumption (between 200 and less than 700 ml/d); high SSB consumption (700 ml/d and more)

Weight change was categorized in weight increase of one kg or more, weight decrease of one kg or more, and weight stable

^a^Anova test, but for ^c^ that is a Chi-square test

^b^Physical activity level was estimated using the Flemish Physical Activity Questionnaire [9]

Compared to non-consumers, the 18 subjects with a SSB intake of 1 L/day on average in 2002 had a weight gain of 4.3 kg (95% CI: 2.0 kg to 6.7 kg). The weight gain was 0.6 kg (95% CI: -0.6 kg to 1.7 kg) when SSB intake of 1 L/day in 2002 was included in models as a continuous variable. In a multivariable model including age, sex, the best predictors of 10-year weight changes were the weight at baseline (*P* < 0.001), then the change in energy intake (*P* = 0.068). No association was found with SSB intake at baseline (*P* = 0.267) and the 10-year change in SSB intake (*P* = 0.130) (Table 4).

**Table 4:** Linear regression analysis of weight change over 10 years according to baseline values of SSB, energy intake and physical activity

Independent variables	β-coefficients	Standard error	95% CI		*P*-value
			Lower bound	Upper bound	
Model 1					
Constant	9.187	1.698	5.851	12.523	<0.001
Weight 2002 (kg/m^2^)	0.961	0.022	0.918	1.003	<0.001
SSB never	ref				
SSB low vs never	0.152	0.600	−1.027	1.330	0.800
SSB medium vs never	−0.173	0.592	−1.337	0.990	0.770
SSB high vs never	4.349	1.210	1.971	6.726	<0.001
Model 2					
Constant	9.728	2.618	4.585	14.871	<0.001
Weight 2002 (kg/m^2^)	0.960	0.022	0.918	1.003	<0.001
Physical activity level 2002^a^	−0.297	1.023	−2.306	1.713	0.772
SSB never	ref				
SSB low vs never	0.157	0.602	−1.025	1.340	0.794
SSB medium vs never	−0.161	0.597	−1.333	1.012	0.788
SSB high vs never	4.338	1.246	1.891	6.786	0.001
Energy 2002 (unit 40 kcal)	0.001	0.014	−0.026	0.028	0.960
Model 3					
Constant	10.196	2.638	5.014	15.337	<0.001
Weight 2002 (kg/m^2^)	0.957	0.022	0.915	1.000	<0.001
Physical activity level 2002^a^	−0.486	1.030	−2.510	1.538	0.637
Log SSB 2002	0.190	0.198	−0.198	0.578	0.335
Energy 2002 (unit 40 kcal)	0.010	0.014	−0.017	0.037	0.485

*SSB* sugar sweetened beverages

Each model is adjusted for all model-specific variables displayed in the Table plus age and gender

Categorization for SSB consumption in 2002: never SSB; low SSB consumption (less than 200 ml/d); moderate SSB consumption (between 200 and less than 700 ml/d); high SSB consumption (700 ml/d and more)

^a^Physical activity level was estimated using the Flemish Physical Activity Questionnaire [9]

The regression model displayed in Table 5 includes weight, physical activity, SSB and energy intake in 2002, plus variables standing for the residual values derived from least square regressions of measures done in 2012 on measures done in 2002. Associations of borderline statistical significance with weight in 2012 were found for changes in energy intake (*P* = 0.068) and physical activity (*P* = 0.091). The β-coefficient values indicate increasing weight with increasing energy intake and reduction in physical activity over time. There was no significant relationship between changes in weight and changes in SSB intake, Healthy Eating Index and Mediterranean Diet Score (data not shown).

**Table 5:** Linear regressions analysis of weight change over 10 years according to changes in SSB, energy intake and physical activity

Independent variables	β-coefficients	Standard error	95% CI		*P*-value
			Lower bound	Upper bound	
Constant	10.840	2.635	5.664	16.016	<0.001
Weight 2002 (kg)	0.957	0.022	0.914	0.999	<0.001
Physical activity level 2002^a^	−0.945	1.038	−2.984	1.093	0.363
Physical activity level residuals^a^	−1.801	1.063	−3.890	0.288	0.091
Log SSB 2002 (ml/day)	0.220	0.198	−0.169	0.609	0.267
SSB residuals (ml/d)	0.002	0.001	−0.001	0.005	0.130
Energy 2002 (unit 40 kcal /day)	0.014	0.014	−0.013	0.041	0.322
Energy residuals (unit 40 kcal/day)	0.029	0.016	−0.002	0.059	0.068

*SSB* sugar sweetened beverages

Model including all variables in Table plus age and gender

^a^Physical activity level was estimated using the Flemish Physical Activity Questionnaire [9]

## Discussion

The present study shows that in prospective studies on SSB consumption and body weight, the use of exposure at baseline or of changes in exposure during follow-up, as well as different statistical analysis modalities may end up in different results and conclusions. Our analysis of quantitative changes in exposure variables over time (Table 5) mimics the analysis of data from randomized trials that compares changes in weight in a group of subjects with increased (or decreased) sugar intake during the trial duration to a group of subject with no change in sugar intake during the trial duration. In this analysis, it is the change in total energy intake that was mainly associated with increase in weight. In addition, while on average body weight tended to increase over time, there was a concomitant decrease in SSB intake. These two results do not support the hypothesis that obesity would be essentially due to an increase in SSB consumption. Rather, adiposity would mainly be the consequence of an imbalance between energy intake and expenditure. Indeed the consumption of SSB (and intake of other sugary foods or beverage) plays a role in adiposity due their energy content, but this role is similar to that played by other foods and caloric beverages.

When our analysis was based on exposure categories at baseline, the 10-year weight change only increased significantly with the highest category of SSB consumption. In many prospective studies on body weight, the excessive SSB intake by a minority of participants is the main contributor to the positive association between SSB consumption and the outcome. Moreover, exposure categories at baseline are often defined so that the categories with lowest and with highest SSB intake include those subjects with the most extreme exposures. For instance, in a prospective study in Singapore [12] the highest (2 or more drinks per week) and the lowest category (almost never SSB consumption) included 10% and 75% of subjects, respectively. In a prospective study of French school teachers [13], the highest SSB consumption category included participants who consumed 359 mL per week or more, which represented 4% of all subjects in the cohort. This category was the only one associated with a statistically significant multivariable adjusted 30% increase of type 2 diabetes.

In contrast with Mozaffarian et al., Pan et al. and Malik et al. [14–16], we found no relation with weight increase and SSB consumption. However, in contrast with those others, we adjusted our models for total energy intake and for baseline SSB consumption. Moreover, Mozaffarian et al. and Pan et al. analyzed data from cohorts from the United States, where, in contrast with Europe, SSB are sweetened with high fructose corn syrup, with possible different satiety and physiological health effects [17].

Many observational studies do not consider changes in food, energy intake and physical activity during the observation period, thus assuming that dietary and energy intakes as well as energy expenditures are stable during follow-up. Our results show that changes in intakes during follow-up do not necessarily depend on amounts consumed at baseline. We thus contend that the results of many prospective studies would have been different if changes in exposures over time had been considered, and if adjustments for changes in energy intake and physical activity had been made. The consequences of this variability in observational results was emphasized by Tai et al. [18]. Using data from the Nurses’ Health Study, the authors illustrated that published observational hypotheses were not associated with a causal confirmation in the intervention design. For three selected health outcomes, i.e., breast cancer, ischaemic heart disease and osteoporosis, the concordance between the results of the Nurses’ Health Study and randomized controlled trials were recorded. For breast cancer, the concordance was 6/24 (25%), for ischaemic heart disease 2/19 (11%) and for osteoporosis 1/5 (20%).

A limitation of the present study is the limited sample size that led to results of borderline statistical significance. Secondly, of the original 1569 participants in 2002, 36% returned for participation in 2012. However, this participation rate is comparable with the proportions of cohort subjects included in statistical analyses of major prospective studies. For instance, Pan et al. [15] included in their analyses 40 to 45% of the participants of the Nurses’ Health Studies and the Health Professional Follow-up Study. However, the aim of our study was not statistical inference of our results to the general population, but to examine the consequences of the variability in ways exposure data are reported and analyzed. For this reason, the representativeness of the used sample is of less priority. A second limitation of the present study is that nutritional exposure was measured with three days food records in 2002 and in 2012. Two or more non-consecutive administrations are required to estimate usual dietary intake distributions [19].

This cohort strongly indicates that high SSB consumption is associated with a clustering of unhealthy behaviours: the highest SSB consumption in 2002 was associated with the highest total energy intake (with or without SSB included in energy calculations), with more smoking, and with the lowest scores for Healthy Eating Index and Mediterranean Diet Score. In other studies too, a high consumption of SSB has been associated with a less healthy dietary pattern and with less healthy lifestyle habits, like for instance, more frequent fast food consumption, higher energy intake, lower physical activity and lower consumption of fruits and vegetables [20–22]. For instance, SSB intake was measured three times in a year in a cohort of 380 US children using food-frequency questionnaires [23]. The authors detected a small group of high SSB consumers, with more than 500 ml a day (*n* = 46, 12%). This group was the most sedentary, was the most eligible for reduced-price school meal, and had the highest energy intake (1799 kcal/day compared to 1021 kcal/day for non-consumers).

Most prospective studies have collected data on foods and beverages other than SSB intake. However, most did not report results or toned down the possible associations between outcomes and the consumption of beverages and foods other than SSB, or with other risk factors like physical inactivity, television watching or fast-food consumption. This selective reporting, coupled with lack of data concerning behaviours associated with SSB consumption, make it difficult to evaluate the exact role of SSB consumption in weight gain. The clustering of unhealthy dietary habits and lifestyles suggests the existence of an obesogenic behaviour that is the simultaneous presence of risk factors for obesity and other metabolic disorders in the same individuals. Because of the clustering of SSB consumption with a more unhealthy diet and behaviours, observational studies can hardly disentangle the specific influence that each dietary item or lifestyle may have on outcomes.

This obesogenic behaviour implies that acting on a specific factor for preventing or treating weight gain is likely to fail if other factors are left unchanged. For instance, if all efforts are concentrated on the reduction of SSB intake, subjects are likely to adopt compensatory behaviors that will cancel out the benefits expected from reduced SSB intake. In this logic, a reductive single food approach would be poorly effective for preventing adiposity. As a matter of fact, many intervention studies that aimed at reducing weight gain via programmes focusing on SSB intake only obtained modest weight loss [24–27].

## Conclusion

In conclusion, results of prospective studies on SSB intake and body weight depend much on the way exposure categories are formed, on whether only data collected at baseline are used, and on the inclusion of changes in energy intake in statistical analyses.

## Abbreviations

ANOVA: Analysis of variance
BMI: Body mass index
CI: Confidence interval
FPAQ: Flemish physical activity questionnaire
PAL: Physical activity level
SD: Standard deviation
SPSS: Statistical package for the social sciences
SSB: Sugar-sweetened beverages
WHO: World Health Organization
